# First person – Daisy Pineda-Suazo

**DOI:** 10.1242/bio.061718

**Published:** 2024-09-25

## Abstract

First Person is a series of interviews with the first authors of a selection of papers published in Biology Open, helping researchers promote themselves alongside their papers. Daisy Pineda-Suazo is first author on ‘ Evaluation of *Octopus maya* enzyme activity of the digestive gland and gastric juice’, published in BiO. Daisy is a postdoc in the Applied Ecophysiology laboratory at Universidad Nacional Autónoma de México, Sisal, México, investigating bioinformatics, molecular biology, omics sciences, biochemistry.


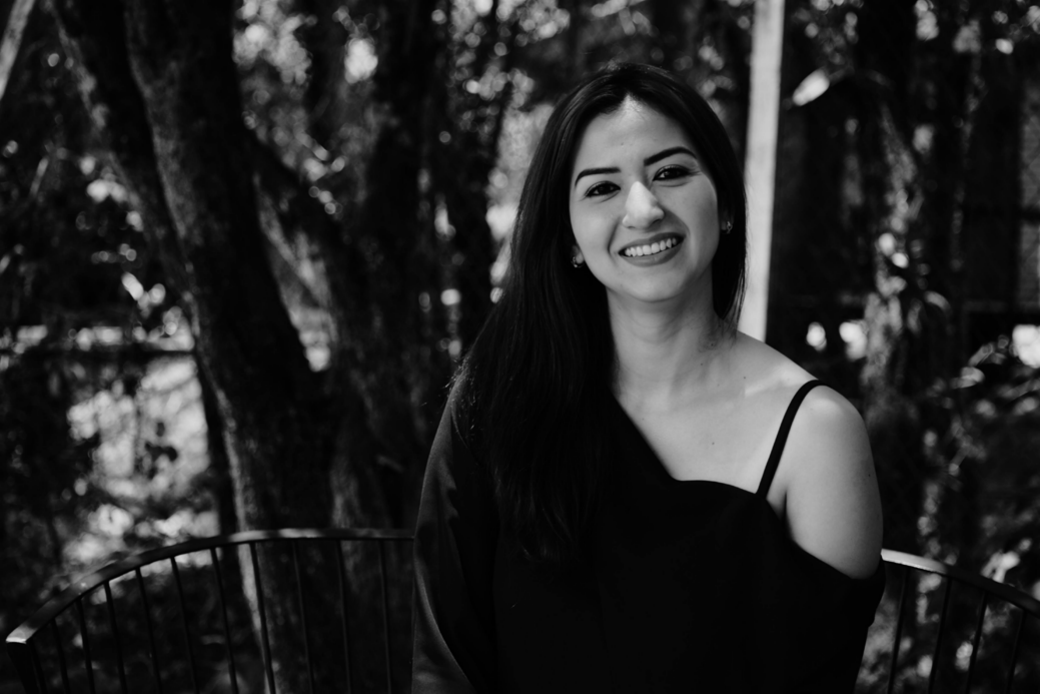


**Daisy Pineda-Suazo**

**Describe your scientific journey and your current research focus**

The objective of the research project in which I am currently working is to discover the transgenerational effects of temperature on digestive capacity through the characterization of the enzymes that make up the digestive system of *Octopus maya*.

**Who or what inspired you to become a scientist?**

Since I was a child, I watched television programs featuring divers and scientists in charge of monitoring wildlife. Those images captivated me and motivated me to become the scientist I am today.

**How would you explain the main finding of your paper?**

With this study we were able to identify some of the enzymes (which are like little tools that help convert food into smaller parts that the body can use) involved in the digestion of *Octopus maya*.

**What are the potential implications of this finding for your field of research?**

This research provides valuable insights into *O. maya* digestive enzyme functions representing a significant advancement in formulating diets crucial for successful octopus farming that may help to fully understand its physiology.

**Figure:**
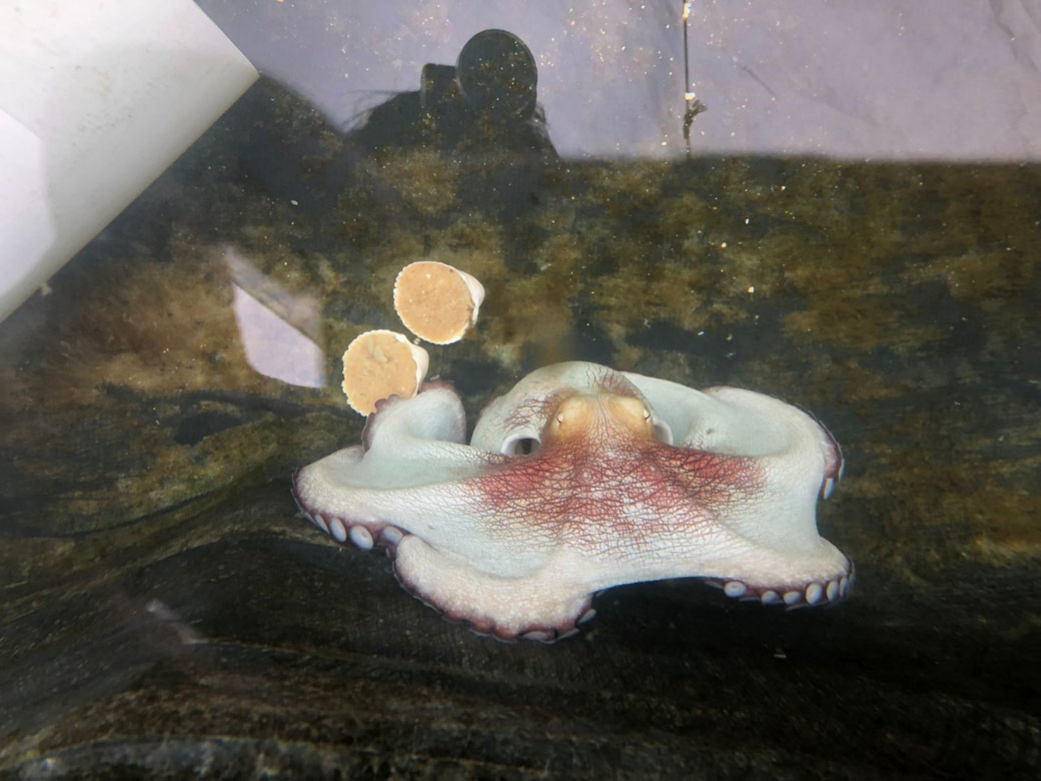
***Octopus maya*** **: treasure of the deep.**

**Which part of this research project was the most rewarding?**

This research is a link in a chain of studies that will allow us to understand the digestive physiology of *O. maya* in order to formulate diets that allow its breeding in captivity, which will contribute to the conservation of the wild population.

**What do you enjoy most about being an early-career researcher?**

As an early-career researcher, I find it fascinating to explore new and diverse topics that allow me to define my own line of research.

**What piece of advice would you give to the next generation of researchers?**

I would advise them to see research as a game in which they can learn, have fun and contribute to solving society's problems. If you manage to fall in love with science, your ideas will flow; on the contrary, if you become a scientific mercenary, you will become frustrated.

**What's next for you?**

Currently I continue working on the identification of the digestive enzymes of *O. maya* through the use of bioinformatics tools as well as the analysis of the microbiome. In addition, I am dedicated to the preparation of audiovisual material that allows the dissemination of the work we do in the Applied Ecophysiology laboratory.
